# *FNDC5* expression and circulating irisin levels are modified by diet and hormonal conditions in hypothalamus, adipose tissue and muscle

**DOI:** 10.1038/srep29898

**Published:** 2016-07-19

**Authors:** B. M. Varela-Rodríguez, L. Pena-Bello, P. Juiz-Valiña, B. Vidal-Bretal, F. Cordido, S. Sangiao-Alvarellos

**Affiliations:** 1 Department of Medicine, School of Health Science, University of A Coruña, Campus de Oza, 15006, Spain; 2 Instituto de Investigación Biomédica de A Coruña (INIBIC), Xubias de Arriba, 84. 15006, A Coruña, Spain; 3 Division of endocrinology, Complexo Hospitalario Universitario de A Coruña (CHUAC), 15006 A Coruña, Spain

## Abstract

Irisin is processed from fibronectin type III domain-containing protein 5 (FNDC5). However, a controversy exists concerning irisin origin, regulation and function. To elucidate the relationship between serum irisin and *FNDC5* mRNA expression levels, we evaluated plasma irisin levels and *FNDC5* gene expression in the hypothalamus, gastrocnemius muscle and different depots of adipose tissue in models of altered metabolism. In normal rats, blood irisin levels diminished after 48-h fast and with leptin, insulin and alloxan treatments, and serum irisin concentrations increased in diabetic rats after insulin treatment and acute treatments of irisin increased blood insulin levels. No changes were observed during long-term experiments with different diets. We suggested that levels of circulating irisin are the result of the sum of the irisin produced by different depots of adipose tissue and skeletal muscle. This study shows for the first time that there are differences in *FNDC5* expression depending on white adipose tissue depots. Moreover, a considerable decrease in visceral and epididymal adipose tissue depots correlated with increased *FNDC5* mRNA expression levels, probably in an attempt to compensate the decrease that occurs in their mass. Hypothalamic *FNDC5* expression did not change for any of the tested diets but increased with leptin, insulin and metformin treatments suggesting that the regulation of central and peripheral *FNDC5*/irisin expression and functions are different.

Metabolic syndrome is characterized by a series of disorders such as, obesity, insulin resistance, glucose intolerance and hyperlipidemia. Obesity is a driver of metabolic syndrome and has emerged as one of the most important medical problems of the 21^st^ century that is a major risk factor for the development of cardiovascular diseases and type 2 diabetes (T2D). According to a World Health Organization estimate, diabetes mellitus affects 346 million people worldwide^1^ and its prevalence continues to increase due to advancing age, increasing obesity rates and inactivity^2^,^3^.

Obesity is a very complex phenomenon in which plays very important roles peripheral tissues such as adipose tissue, liver and muscle as well the neurohormonal and neurotransmitter dysregulation^4^,^5^,^6^,^7^,^8^. In this regard, the hypothalamus plays a key role in energy homeostasis. It controls food intake and senses multiple circulating peripheral signals such as leptin, insulin, adiponectin, gut hormones and nutrients that function as “indicators” that interact with specific hypothalamic regions to disturb the energy balance both peripherally and centrally^9^,^10^,^11^.

In 2012, Boström and colleagues discovered that in muscle, exercise increases the expression of FNDC5 (Fibronectin type III domain-containing protein 5), a membrane protein encoded by the *FNDC5* gene. The FNDC5 protein is cleaved and secreted as a new hormone called irisin^12^, suggesting that some of beneficial effects of exercise could be mediated by this hormone. Irisin induces the browning of white adipose tissue (WAT), thereby increasing thermogenesis and possibly improving glucose homeostasis^12^,^13^,^14^. There have been many studies attempting to correlate plasma irisin levels with metabolic disorders such as obesity, diabetes, non-alcoholic fatty liver disease (NAFLD) and polycystic ovary syndrome (PCOS) (both associated with metabolic syndrome), however, the results were not consistent among the various studies (reviewed by^15^). Recently, it has been reported that irisin is also produced by adipocytes^14^,^16^ and that it is present in human cerebrospinal fluid (CSF), although its role in the brain is largely unknown. FNDC5 inhibition reduces neurogenesis^17^, while its overexpression stimulates neural differentiation^18^, and pharmacological doses of irisin increase the proliferation of mouse hippocampal neuronal cells^19^ similar to the effects of endurance exercise, a process associated with the increased expression of brain derived neurotrophic factor (BDNF)^20^. In the hypothalamus, irisin appears colocalized with neuropeptide Y (NPY) in the neuronal cells of the paraventricular nucleus^21^. These findings support the idea that irisin has both peripheral and central functions. Despite the promising results, many aspects of the regulation, secretion and physiological aspects of irisin remain unknown. Some previous studies even questioned the existence of irisin^22^, although that point was clarified recently by Jedrychowski and coworkers^23^ who showed that human irisin exists, circulates, and is regulated by exercise.

The main aim of this study was to test the hypothesis that metabolic status regulates both central and peripheral *FNDC5* mRNA expression levels and irisin synthesis and secretion. Most of the published works studied the effects of irisin on browning in subcutaneous adipose tissue, but few did so on other fatty deposits^16^. To elucidate the relationship between serum irisin and *FNDC5* mRNA expression levels, we evaluated *FNDC5* mRNA expression levels in the hypothalamus, gastrocnemius muscle, brown adipose tissue and visceral (abdominal), epididymal and subcutaneous adipose tissue. We used different models of altered metabolism: rats reared with a high-fat diet (HFD) or caloric restriction (CR) for three months, fasting, and treatments with leptin, insulin, metformin, alloxan and alloxan plus insulin.

## Materials and Methods

### Animals

All experiments and animal protocols involved in this study were reviewed and approved by the Ethics Committees of the University of A Coruña and CHUAC, in accordance with EU Normative for the use of experimental animals. Wistar rats of different ages used in this study were housed in a temperature controlled room with a 12 h light, 12 h dark cycle (lights from 08:00 to 20:00 h). To study the expression of profile of *FNDC5* mRNAs we used females to obtain ovaries and placenta samples in the remaining experiments we used males. All rats were provided with *ad libitum* access to water.

### Body composition and tissue dissection

Liver and visceral and epididymal WAT were dissected and weighed, and somatic indices were calculated as the ratio between tissue and body weights. Rats were killed by cervical dislocation and trunk blood was extracted. The hypothalami were dissected and stored at −80 °C until further processing for real-time PCR assays. The hypothalamus was defined by the posterior margin of the optic chiasm and the anterior margin of the mammillary bodies to a depth of approximately 2 mm, following previous protocols^24^.

### Quantitative real-time PCR

Total RNA was extracted from tissues using TRIzol reagent (Invitrogen). RNA quality and concentrations were determined by agarose gel electrophoresis and spectrophotometry in a ND-1000 NANODROP 385 spectrophotometer (Thermo-Scientific), respectively. Real-time PCR was performed on a Roche LightCycler 480 Real Time PCR Detection System. For mRNA quantification, 1 μg of total RNA per tissue sample was treated with RQ1 RNAse-free DNAse-I (Promega) and retro-transcribed (RT) in a 30 μl reaction, using M-MLV reverse transcriptase and random primers (Invitrogen). For PCR, we used SYBR Green qPCR Master Mix (Roche). The primers used were: *HPRT* forward 5′-AGCCGACCGGTTCTGTCAT-3′; *HPRT* reverse 5′-GGTCAATAACCTGGTTCATCATCAC-3′; *FNDC5* forward 5′-GAGGTGCTGATCATCGTCGT-3′; *FNDC5* reverse 5′-GAGCAAGCACTGAAAGGGTTT-3′; *SOCS3* forward 5′-ACCACTACATGCCGCCCCCA-3′; *SOCS3* reverse 5′-TCGGCTCAGTACCAGCGGGA-3′; *UCP1* forward 5′-CAATGACCATGTACACCAAGGAA-3′; *UCP1* reverse 5′-GATCCGAGTCGCAGAAAAGAA-3′. For data analysis, the input value of the target gene was standardized to the *HPRT* value for each sample. PCR was initiated by one hold of 95 °C for 10 min, followed by 40 cycles of 15 s at 95 °C, 55 s at 60 °C, and 5 s at 72 °C, followed by one hold of 72 °C for 10 min.

### Plasma measurements

Irisin and insulin levels were measured in plasma samples by commercially available ELISA kits according to the manufacturer’s specifications (Adipogen and DRG respectively). Intra- and inter- assay coefficients of variations for ELISAs were below 8% and 10% (irisin) and 3% and 5% (insulin) respectively.

### Experimental design

#### Tissue distribution and differential expression of the FNDC5 gene in rats

The expression of profile of *FNDC5* mRNAs was explored in a broad panel of tissues from adult (>75 days-old, n = 3) male and female rats. The tissues were removed, quick frozen and stored at −80 °C until used for RT-PCR expression analyses.

#### Alterations in FNDC5 gene expression and circulating irisin levels in rats reared with different diets: Long-term experiments

In order to define the hypothalamic, skeletal muscle and brown and white adipose tissue *FNDC5* mRNA profiles and serum irisin levels and their possible variation with different body weights, % fat mass, insulin sensitivity and other obesity-related factors, 30 male rats were used. At weaning day (postnatal day 21), the animals were randomly divided into three groups: (*1*) control group, with *ad libitum* access to standard chow diet (ND = normal diet) (3.85 kcal/g; 10% kcal % fat; *Research Diets, Inc*.) (n = 10); (*2*) diet induced obesity (DIO) group, with *ad libitum* access to HFD diet (4.73 kcal/gm; 45% kcal % fat; *Research Diets, Inc*.) (n = 10); and (*3*) CR group (n = 10), with standard chow diet but the food was restricted to 65% of the daily amount ingested by the control group. To this CR group, a fixed amount of food was provided daily, and the animals ate all the food offered. The animals subjected to this feeding regimen had a lower percentage of body fat and increased sensitivity to insulin. The animals were housed under these feeding regimens for three months until they were sacrificed. Animals were sacrificed two hours after the beginning of the light phase at 10:00 a.m.

#### Alterations in FNDC5 gene expression and circulating irisin levels by fasting: Short-term experiments

We evaluated the impact of caloric restriction under a short-term duration. To this end, adult rats (BW: 260 g ± 4, 11 weeks of age) were fed with ND and subjected to fasting for 48 hours.

#### Effects of leptin in FNDC5 gene expression and circulating irisin levels in fed and fasted rats

To monitor changes in *FNDC5* mRNA expression and circulating irisin in conditions of altered leptin signalling, adult rats (350 g ± 9) were fed *ad libitum* and received two intraperitoneal (IP) injections of leptin (*Prospec,* 1 mg/kg dissolved in 200 μl of saline) or saline (control group). Injections were applied 24-h and 2-h prior to animal sacrifice. Treatments started at 08:00 a.m. and were carried out in the light phase.

In an attempt to establish if leptin regulates hypothalamic and peripheral *FNDC5* mRNA expression levels and plasma irisin levels in a nutritional-dependent fashion, we fed *ad libitum* one group of adult rats (189 g ± 9) and another group was deprived of food for 48 h. Fed (with normal values of insulin, leptin and glucose) and fasted (state associated with low levels of insulin, glucose and leptin) rats received two IP injections of leptin (*Prospec,* 1 mg/kg dissolved in 200 μl of saline) or vehicle. Injections were applied at 24-h and 48-h after the beginning of fasting. Treatments started at 08:00 a.m. and were carried out in the light phase. Animals were sacrificed 2-h after the 2^nd^ injection (10:00 a.m.).

#### Effects of insulin and metformin in FNDC5 gene expression and circulating irisin levels

To evaluate the impact of insulin and insulin sensitizers in *FNDC5* mRNA expression and plasma irisin levels, we treated rats fed with ND with insulin or metformin for 2 weeks. To this end, adult rats (290 g ± 2.2) were fed *ad libitum*. Insulin (*Novo Nordisk Phama*, 1 UI/kg, dissolved in 200 μl of saline and administrated IP), metformin (*Sandoz*, 300 mg/kg, dissolved in 200 μl of saline and administrated subcutaneously) and vehicle treatments were carried out in the light phase. Animals were sacrificed 2 h after the last injection (day 15).

#### Effects of irisin in serum insulin and glucose levels in fed rats

To monitor changes in circulating insulin and glucose levels in conditions of altered irisin, adult rats (197 g ± 3.5) were fed *ad libitum* and received two intraperitoneal (IP) injections of irisin (Phoenix, 20 μg/rat dissolved in 200 μl of saline) or saline (control group). Injections were applied 24-h and 2-h prior to animal sacrifice. Treatments started at 09:00 a.m. and were carried out in the light phase.

#### Effects of diabetes on FNDC5 gene expression and circulating irisin levels in fed and fasted rats

In order to assess the effect of diabetes on *FNDC5* mRNA expression and circulating irisin levels, two new experiments were conducted in which we induced diabetes in adult rats (274 g ± 3.2) by a single injection of alloxan (Sigma-Aldrich, 130 mg/kg dissolved in 200 μl of saline and administrated IP). In one of them, six days after alloxan injection a group of rats was fasted for 48 h to reduce glucose levels. The animals were sacrificed 8 days after alloxan injection.

In the other experiment, nine days after alloxan injection part of the rats were treated with subcutaneous injections of exogenous human NPH insulin to maintain glycemia in these animals as close to the normoglycemia as possible. The glucose levels were measured every day at 12:00 h and 20: 00 h with a glucometer and a blood drop from the tail vein. The normal dose of NPH insulin was 9.5 U/day but the daily dose of insulin was adjusted according to the glycemia of each animal. Two units of insulin were injected at 13:00 h and the 7.5 remaining units at 21:00 h. The control group received saline solution. The animals were sacrificed 8 days after insulin treatment.

### Statistical analysis

Data were analysed using SigmaStat 3.1 (Systat Software, Inc.) and are presented as the means ± SEM. Statistical significance was determined by t-tests (experiments with two groups), one-way ANOVA with *post hoc* Tukey’s tests (experiments with more than two groups and one variable) or two-way ANOVA with *post hoc* Tukey’s tests (experiments with more than two groups and two variables). *P* < 0.05 was considered significant. Different letters or symbols above bars indicate statistical significance.

## Results

### **Tissue distribution and differential expression of the *FNDC5*** **gene in rats**

**Figure 1:**
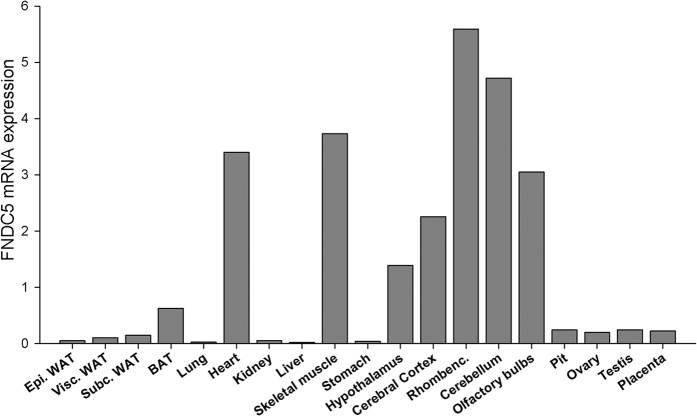
Expression profile of the *FNDC5* gene in different tissues in the adult rat. A panel of tissues from adult male and female (ovary and placenta) rats was screened for expression of *FNDC5* mRNAs, using real-time RT-PCR.

As shown in Fig. 1, *FNDC5* mRNA is prominently expressed in different brain areas and muscle (both cardiac and skeletal). Brown adipose tissue, pituitary, placenta, testis and ovary also showed relatively high expression levels with modest expression in the depots of white adipose tissue. Other tissues such as lung, kidney, liver, and stomach had very low or undetectable *FNDC5* mRNA levels.

### Alterations in *FNDC5* gene expression and circulating irisin levels in rats reared with different diets: Long-term experiments

**Figure 2:**
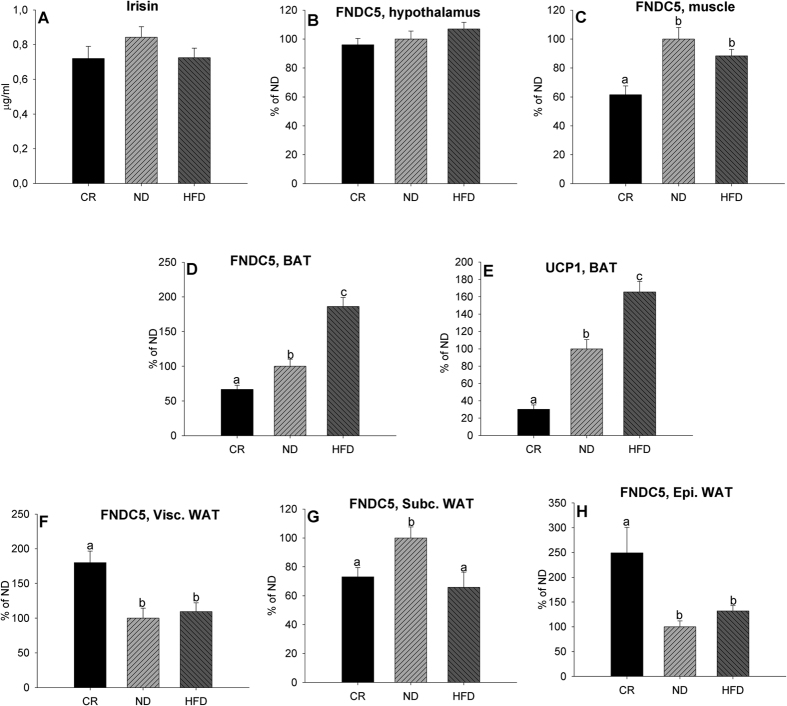
Effect of the HFD and CR for three months on plasma irisin (**A**) levels, *FNDC5* expression levels in the hypothalamus (**B**), muscle (**C**), BAT (**D**) and visceral (**F**), subcutaneous (**G**) and epididymal (**H**) white adipose tissue of adult male rats and *UCP1* expression levels in BAT (**E**). Values are expressed in μg/ml for circulating irisin and arbitrary units for *FNDC5* and *UCP1* gene expression, as the mean ± SEM, where ND = 100%. Different letters above the bars indicate statistical differences (One-way ANOVA with post hoc Tukey’s test, a value of P < 0.05 was considered statistically significant). CR: caloric restriction (n = 10), ND: normal diet (n = 10) and HFD: high fat diet (n = 10). The animals were reared on these diets for three months after weaning.

Corporal and some plasmatic parameters of rats reared for three months with different diets were published previously^25^. The % fat mass was very low in animals reared in CR (scarcely a 2%) and increased to 9.5% in the control group and 18.8% in animals with HFD (Table 1). The animals subjected to CR displayed signs of increased insulin sensitivity because they had the lowest basal glucose and insulin levels. In contrast, HFD rats presented signs of insulin resistance and metabolic deregulation, as evidenced by higher basal glucose, insulin, and leptin and increased plasma triglyceride concentrations (Table 1)^25^. To monitor if metabolic alterations induced by the different diets influence circulating irisin levels and *FNDC5* gene expression, we analysed serum irisin values and *FNDC5* mRNA expression by real-time RT-PCR. As shown in Fig. 2A, animals subjected to CR and HFD displayed diminished serum irisin levels but the differences were not statistically significant. No changes were observed in hypothalamic (Fig. 2B) *FNDC5* mRNA expression. The expression profiles of *FNDC5* in muscle (Fig. 2C) and inguinal subcutaneous WAT (Fig. 2G) were similar to the circulating levels of irisin, with lower values in the CR in both tissues and in subcutaneous WAT in animals fed with HFD. Levels of *FNDC5* and *UCP1* (uncoupling protein 1) gene expression in BAT (Fig. 2D,E) increased in proportion to the percentage of fat mass and *FNDC5* gene expression was enhanced with CR in visceral (Fig. 2F) and epididymal (Fig. 2H) WAT.

### Alterations in *FNDC5* gene expression and circulating irisin levels in fasted rats: short-term experiments

Main body parameters and plasma levels of key factors in rats fasted for 48 h are shown in Table 2. As expected, BW gain and glucose, insulin, and triglyceride plasma levels diminished with fasting. The epididymal-somatic index did not change with the fast, while the percentage of visceral fat and hepatic somatic index decreased in fasted rats (Table 2).

**Figure 3:**
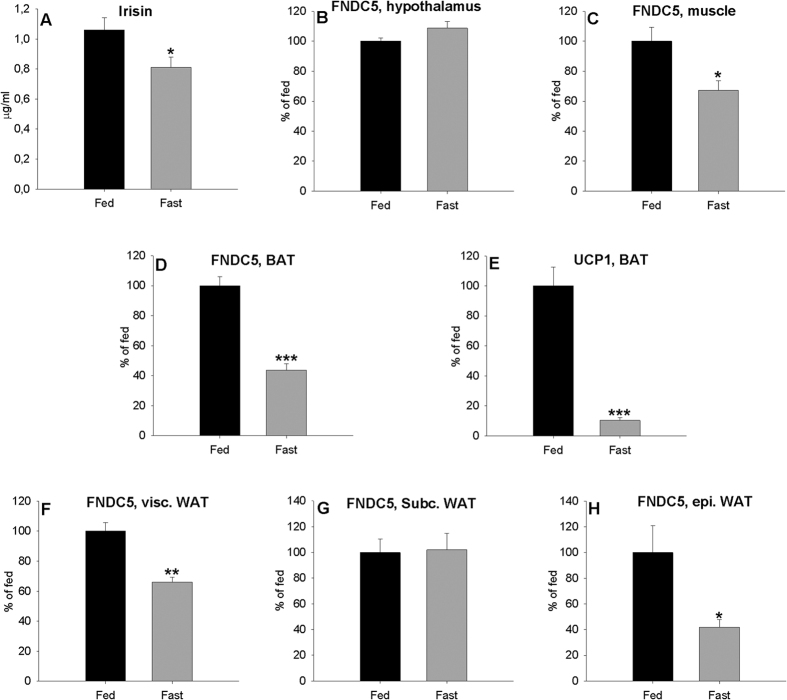
Effect of 48 h of fasting on plasma irisin (**A**) levels, *FNDC5* expression levels in the hypothalamus (**B**), muscle (**C**), BAT (**D**) and visceral (**F**), subcutaneous (**G**) and epididymal (**H**) white adipose tissue and UCP1 expression levels in BAT (**E**) of adult male rats. Values are expressed in μg/ml for circulating irisin and arbitrary units for *FNDC5* and *UCP1* gene expression, as the mean ± SEM, where fed = 100%. N = 6. Different symbols above the bars indicate statistical differences (t-test, a value of P < 0.05 was considered statistically significant). *P < 005 vs. fed.

The results *FNDC5* mRNA expression and circulating irisin levels during fasting are shown in Fig. 3. Interestingly, a 48-h fast led to a decrease in serum irisin levels (Fig. 3A) and reduced *FNDC5* mRNA levels in muscle (Fig. 3C), BAT (Fig. 3D) and in visceral and epididymal white adipose tissue (Fig. 3F,H). Values of *FNDC5* mRNA in subcutaneous WAT did not change (Fig. 3G). *UCP1* expression levels in BAT (Fig. 3E) decreased significantly with fasting, just like did *FNDC5* values. In the hypothalamus, the effect of fasting was contrary although the difference was not statistically significant (*P* > 0.05) (Fig. 3B). These results suggest that fasting has different effects on central and peripheral *FNDC5* gene expression.

### Effects of leptin on *FNDC5* gene expression and circulating irisin levels

**Figure 4:**
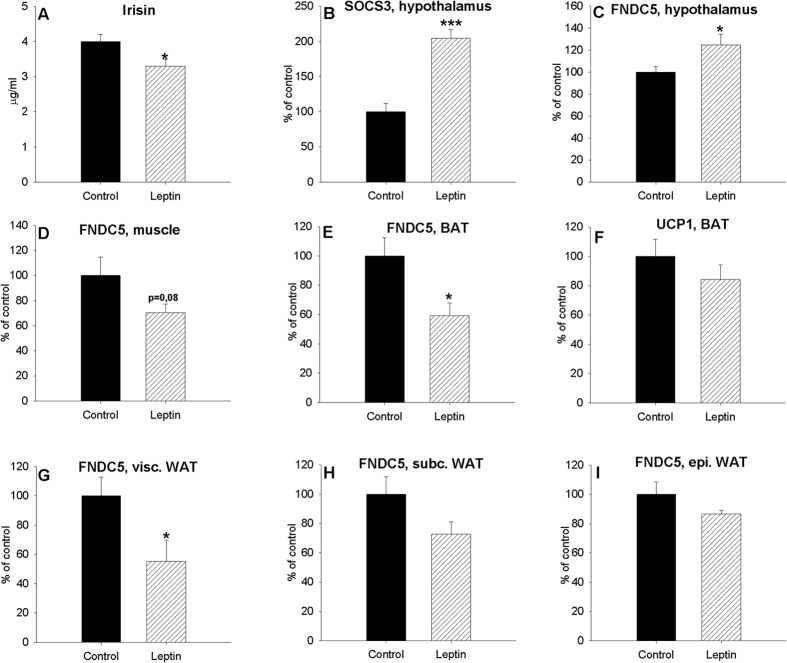
Effect of 2-day IP leptin treatment in fed animals on plasma irisin (**A**) levels, *SOCS3* gene expression (**B**), *FNDC5* expression levels in the hypothalamus (**C**), muscle (**D**), BAT (**E**) and visceral (**G**), subcutaneous (**H**) and epididymal (**I**) white adipose tissue of adult male rats and *UCP1* expression levels in BAT (**F**). Values are expressed in μg/ml for circulating irisin and arbitrary units for *FNDC5* and *SOCS3* gene expression, as the mean ± SEM, where control = 100%. n = 6–7. *, ***P < 0.05 and 0.01 respectively vs. control (rats injected with saline) (t-test).

The efficiency of the treatment was corroborated by the increased expression of hypothalamic *suppressor of cytokine signalling 3 (SOCS3*) (Fig. 4B), a primary target of leptin with a negative feedback regulation^26^. Interestingly, leptin treatment was associated with increased hypothalamic *FNDC5* expression levels (Fig. 4C) but decreased serum irisin levels (Fig. 4A) like muscle (Fig. 4D), BAT (Fig. 4E) and visceral (Fig. 4G), subcutaneous (Fig. 4H) and epididymal (Fig. 4I) white adipose tissue *FNDC5* gene expression. These results suggest that leptin has different effects on central and peripheral *FNDC5* gene expression, which is similar to the effects of fasting. Unlike *FNDC5* gene expression, no changes were observed in BAT *UCP1* mRNA expression levels after treatment with leptin (Fig. 4F).

**Figure 5:**
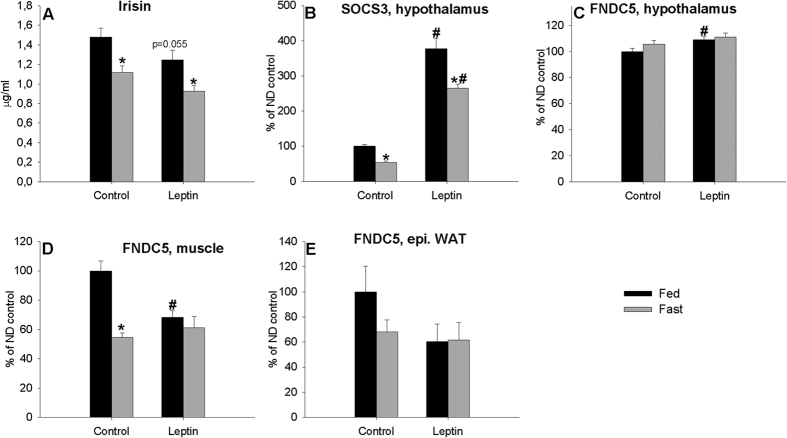
Effect of 2-day IP leptin treatment in fed and fasted animals on plasma irisin levels (**A**), *SOCS3* gene expression (**B**) and *FNDC5* expression levels in the hypothalamus (**C**), muscle (**D**), and epididymal (**E**) white adipose tissue of adult male rats. Values are expressed in μg/ml for circulating irisin and arbitrary units for *FNDC5* and *SOCS3* gene expression, as the mean ± SEM, where fed animals treated with saline were the controls = 100%. n = 6–7. Different symbols above bars indicate statistical differences (Two-way ANOVA with post hoc Tukey’s test, a value of P < 0.05 was considered statistically significant). *P < 005 vs. fed; ^#^P < 0.05 vs. saline.

In order to corroborate the previous results and to assess if leptin regulates hypothalamic and peripheral *FNDC5* mRNA expression levels and plasma irisin levels in a nutritional-dependent fashion we repeated the last experiment but in fed and fasted rats. The results are showed in Fig. 5.

Overall, there was a significant (*P* < 0.001 fed *vs*. fast and *P* = *0.031* vehicle *vs*. leptin, two-way ANOVA) effect of fasting and leptin treatment on plasma irisin levels but no significant (*P* > 0.05, two-way ANOVA) effect of leptin treatment × *status* nutritional interaction, although the comparisons for leptin treatment within fed animals had a P = 0.055, similar to the diminished irisin levels in fasting animals in the previous experiment (Fig. 5A). In the hypothalamus, *SOCS3* gene expression increased in all animals treated with leptin and diminished with fasting (Fig. 5B). In concordance with the previous results, *FNDC5* mRNA expression levels diminished in muscle tissue of animals treated with leptin but this effect disappeared with fasting (Fig. 5D). Similar results were obtained for the hypothalamus, where *FNDC5* mRNA expression levels increased in fed animals treated with leptin; however, this effect was not present in fasted animals (Fig. 5C).

#### Effects of insulin and metformin on FNDC5 gene expression and circulating irisin levels

**Figure 6:**
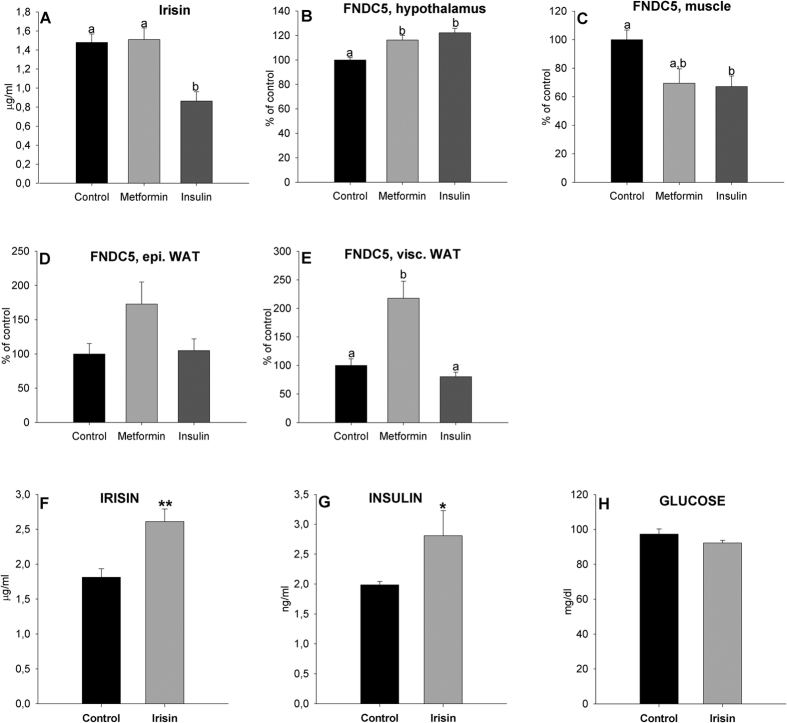
Effect of a 14-day IP saline and insulin and subcutaneous metformin treatment on plasma irisin levels (**A**) and *FNDC5* expression levels in the hypothalamus (**B**), muscle (**C**) and epididymal (**D**) and visceral (**E**) WAT of adult male rats and effect of 2-day IP irisin treatment in fed animals on plasma irisin (**F**), insulin (**G**) and glucose levels (**H**). Different letters above the bars indicate statistical differences (One-way ANOVA with post hoc Tukey’s test, a value of P < 0.05 was considered statistically significant). ***P < 0.05 and 0.01 respectively vs. control (rats injected with saline) (t-test). Values are expressed as the mean ± SEM where animals treated with saline. N = 6 (metformin treatment), 8 (saline and insulin treatments) and 7 (irisin treatment). Values are expressed in mg/dl for plasma glucose levels, ng/ml for plasma insulin levels and μg/ml for circulating irisin levels. Arbitrary units were used for *FNDC5* gene expression where rats injected with saline = 100%.

As expected, insulin and metformin injections diminished glucose levels demonstrating their efficacy (Table 3). Insulin and metformin treatments provoked a lower body weight gain with respect to controls; however, epididymal and visceral WAT mass were unchanged in insulin treated rats. With metformin, epididymal somatic index diminished with respect to controls and insulin treated rats. The hepatosomatic index and plasma triglyceride levels were reduced in insulin-treated rats (Table 3). Plasma irisin levels diminished in animals treated with insulin (Fig. 6A). Hypothalamic *FNDC5* mRNA expression levels increased with insulin and metformin treatments (Fig. 6B). Muscle *FNDC5* mRNA expression levels diminished in rats treated with insulin and metformin with respect to their controls, although with metformin the differences were not statistically significant (Fig. 6C). WAT *FNDC5* gene expression increased with metformin (Fig. 6D,E), however in epididymal WAT (Fig. 6D) the differences were again not significant.

### Effects of irisin in serum insulin and glucose levels in fed rats

Exogenous irisin treatment caused a physiologic increase in circulating irisin levels (Fig. 6F). In turn, irisin caused an increase in the amount of circulating insulin (Fig. 6G) without altering blood glucose levels (Fig. 6H).

### Effects of diabetes on *FNDC5* gene expression and circulating irisin levels

**Figure 7:**
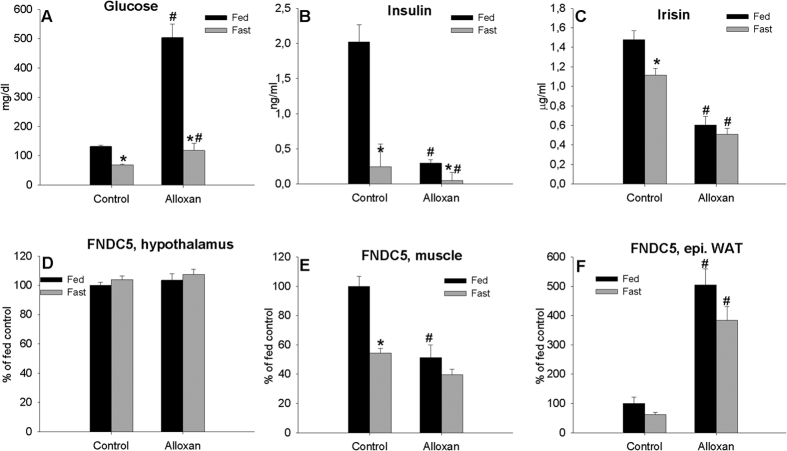
Effect diabetes induced by a single injection of alloxan in fed and fasted (48 h) animals on plasma glucose (**A**) insulin (**B**) and irisin (**C**) levels and *FNDC5* gene expression levels in the hypothalamus (**D**), muscle (**E**) and epididymal (**F**) WAT of adult male rats. *P < 0.05 vs. fed animals. ^#^P < 0.05 vs. saline. (Two-way ANOVA with post hoc Tukey’s test, a value of P < 0.05 was considered statistically significant). Values are expressed in mg/dl for plasma glucose levels, ng/ml for plasma insulin levels and μg/ml for circulating irisin levels. Arbitrary units were used for *FNDC5* gene expression, as the mean ± SEM, where rats fed and injected with saline = 100%. N = 8 (saline treatment) or 10 (alloxan treatment).

As expected, blood glucose levels in both fed and fasted rats treated with alloxan were higher compared with their controls (Fig. 7A). When fed rats showed blood glucose levels higher than 260 mg/dl were classified as diabetic. Insulin levels diminished significantly in animals treated with alloxan and with fasting (Fig. 7B). Alloxan treatment diminished the percentage of visceral and epididymal WAT considerably both in fed and fasted rats (data not shown). Plasma irisin levels diminished with alloxan in fed and fasted animals with respect to their controls (Fig. 7C). Fasting was also associated with reduced circulating levels of irisin (as we observed in experiment 3), but only in control animals (Fig. 7C). The *FNDC5* gene showed a similar expression profile in muscle (Fig. 7E). However, epididymal WAT *FNDC5* mRNA expression levels increased with alloxan treatment in fed and fasted rats (Fig. 7F). No differences were observed in hypothalamic *FNDC5* gene expression (Fig. 7D). Again, muscle *FNDC5* mRNA expression levels diminished with fasting but only in control rats (Fig. 7E). Diabetes also diminished *FNDC5* gene expression but this effect was dependent of nutritional *status.* Independently of feeding, WAT *FNDC5* gene expression increased substantially with alloxan treatment (Fig. 7F).

**Figure 8:**
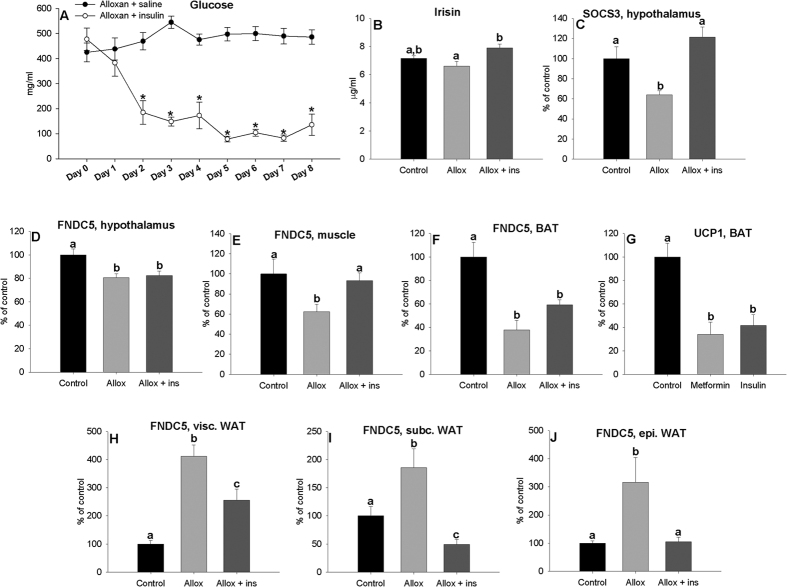
Effect of treatment of diabetic rats using 9.5 IU/day of NPH insulin for 8 days on plasma glucose levels (**A**) and effect after 8-day treatments of diabetics with insulin on plasma irisin (**B**) levels and *SOCS3* (**C**), *FNDC5* expression levels in the hypothalamus (**D**), and *FNDC5* expression levels in muscle (**E**), BAT (**F**) and visceral (**H**), subcutaneous (**I**) and epididymal (**J**) white adipose tissue of adult male rats and *UCP1* expression levels in BAT (**G**). *P < 0.05 vs. diabetics rats injected with saline. Different letters above the bars indicate statistical differences (One-way ANOVA with post hoc Tukey’s test, a value of P < 0.05 was considered statistically significant). Values are expressed in mg/dl for plasma glucose levels and μg/ml for circulating irisin levels. Arbitrary units were used for *FNDC5* and *SOCS3* gene expression, as the mean ± SEM, where normal rats injected with saline = 100%. N = 8.

In the next experiment, we tested if parameters altered by alloxan-induced diabetes can be reversed by insulin therapy. As in the previous experiment, the alloxan treatment induced a robust increase in blood glucose levels nine days after alloxan injection (Fig. 8A). Glucose levels dropped below 200 mg/dl 48 h after beginning insulin treatment, and after 5 days normoglycemic levels were maintained until the animals were sacrificed (Fig. 8A). No changes were observed in plasma irisin levels of animals treated with alloxan with respect to the control group; however they showed lower levels than the rats subjected to insulin therapy (Fig. 8B). Hypothalamic *SOCS3* (Fig. 8C) and *FNDC5* (Fig. 8D) mRNA expressions decreased in diabetic rats. But unlike *FNDC5* gene expression, *SOCS3* mRNA expression levels were reversed by insulin treatment. *FNDC5* mRNA expression was reduced in muscle (Fig. 8E) and BAT (Fig. 8F) in diabetic rats. *UCP1* gene expression in BAT (Fig. 8G) showed exactly the same pattern that *FNDC5* mRNA expression levels, reinforcing the hypothesis that *UCP1* and *FNDC5* are related. In muscle, the values were recovered by insulin treatment. In all WAT depots studied, *FNDC5* gene expression enhanced with diabetes, and these values decreased significantly (even over control values) after insulin treatment (Fig. 8H–J).

## Discussion

Initially irisin was described as a myokine that induces white fat “browning” *in vivo* and *in vitro*^12^, and later as an adipokine^16^. Since its discovery there have been numerous published studies about its regulation and function. Böstrom *et al*. proposed that FNDC5 was cleaved producing the soluble protein irisin that increases total body energy expenditure and resistance to obesity-linked insulin-resistance, and that this effect was a result of browning produced by irisin in adipose tissue^12^. Both in humans and rodents correlated brown fat tissue formation with anti-obesity effects, suggesting a central role of beige adipocyte thermogenesis in whole-body energy metabolism^27^,^28^,^29^. However, controversy has emerged regarding the association between irisin and obesity or metabolic disorders. Some studies have suggested that irisin ameliorates obesity, glucose disorders and insulin resistance^14^,^30^,^31^ and that circulating irisin is negatively associated with BMI and percentage of fat mass^14^,^32^; however, other studies have found that irisin is positively correlated with these parameters^33^,^34^ while others have revealed no correlation between irisin and BMI^35^,^36^,^37^.

To elucidate the relationship between serum irisin levels and *FNDC5* gene expression in different tissues, we evaluated blood irisin levels and *FNDC5* mRNA expression levels in the hypothalamus, gastrocnemius muscle, brown adipose tissue and different depots of WAT [visceral (abdominal), epididymal and subcutaneous] in different models of altered metabolism and moreover we studied the effects of exogenous irisin on insulin levels.

To begin, we studied the distribution of *FNDC5* mRNA expression in different rat tissues. As previously reported, *FNDC5* mRNA was expressed in high levels in muscles^20^,^34^ and also in all brain regions analysed^18^,^38^ suggesting a role for FNDC5/irisin in the brain. This theory is supported by different reports of FNDC5/irisin function in different brain processes. Endurance exercise enhances *FNDC5* gene expression in the hippocampus of mice which in turn stimulates *BDNF* gene expression and forces expression of *FNDC5* in primary cortical neurons increasing cell survival^20^. In humans, irisin was detected in cerebrospinal fluid and hypothalamic sections, especially paraventricular neurons, colocalising with neuropeptide Y^21^. We also reported high levels of *FNDC5* mRNA expression in BAT, which could be due to the prominent role of BAT in the release of heat through the action of UCP1 and in the control of glucose and lipid metabolism^39^ since cold exposure increases circulating irisin levels^40^ in addition to irisin driving brown-fat-like development of white fat and thermogenesis stimulating *UCP1* expression^12^. In this work, we demonstrated that the *FNDC5* and *UCP1* gene expression patterns are identical in BAT during chronic caloric restriction, fasting, feeding with HFD and treatments with alloxan or alloxan plus insulin. These results are supported by other studies in rodents demonstrating that HFD increases brown adipose tissue UCP1 mRNA and protein content^41^ and that fasting diminishes UCP1 gene and protein expression^42^. We observed that alloxan treatment clearly reduced *FNDC5* expression in BAT, which could be explained by a reduced thermogenic capacity of BAT as noted in animal studies with chronic insulin deficiency^43^,^44^. Furthermore, mice with type 1 diabetes *mellitus* showed a reversion of glucose homeostasis to normalcy increasing BAT quantity by transplants^45^. After acute leptin treatment in fed rats, we observed no differences in BAT *UCP1* mRNA, as is consistent with previous reports^42^,^46^; however, *FNDC5* mRNA expression was reduced in these conditions. Sivitz *et al*. observed that leptin effects on UCP1 mRNA and protein levels may be discordant^42^, therefore it cannot be ruled out that our discrepancies are due to the tendency for UCP1 mRNA and protein expression levels after leptin treatment to be different or even that this also occurs with FNDC5. The relatively high expression of *FNDC5* in the reproductive neuroendocrine axis (hypothalamus, pit, ovary, testis and placenta) suggests an effect of irisin on reproductive function. However, additional studies are required to clarify whether such a relationship exists.

It must be borne in mind that different WAT depots may differ in physiological function. Excess accumulation of visceral adipose in the abdominal cavity is associated with metabolic syndrome, diabetes, cardiovascular disease and mortality^47^. However, in rodents, subcutaneous WAT, appears to be relatively benign and transplantation of subcutaneous adipose tissue from normal^48^ or exercise-trained donor^49^ to visceral cavity can improve glucose metabolism and insulin sensitivity. Irisin may promote maintenance of “healthy” subcutaneous adipose because *FNDC5* is induced by exercise in this depot and increases brown/beige fat cells^12^. Although WAT *FNDC5* gene expression may represent only a small fraction of that expressed in muscle, brain, or even brown adipose tissue, Moreno-Navarrete *et al*. suggested that it is the adipose tissue (studies realized with visceral and subcutaneous WAT) and not the skeletal muscle expression that correlates with levels of circulating irisin in humans^14^. In support, Yang *et al*. demonstrated that FNDC5 protein expression in adipose tissue, not skeletal muscle, contributed to the change of circulating irisin in HFD-induced obese mice^50^; but, Yang *et al*. only analysed subcutaneous abdominal adipose tissue. However, the existence of diverse fat depot states in rats offers the possibility of differential contributions from these depots to circulating irisin levels. To explore this, we analysed *FNDC5* gene expression in epididymal, visceral (abdominal) and subcutaneous WAT and BAT during different states of metabolic distress. We observed that the alterations in blood irisin levels resulting from changes in the feeding depend on the duration and nature of the changes. In long-term experiments, neither caloric restriction nor high fat diet cause changes in the circulating irisin levels, however with 48 hours of fasting, blood irisin levels drop along with the decline observed in *FNDC5* mRNA expression levels in muscle, BAT and visceral and epididymal adipose tissue. However, changes were not observed in subcutaneous fat, suggesting that the changes produced in circulating irisin levels with short-term restriction caloric are not related to *FNDC5* gene expression levels in subcutaneous fat, but could be related to variations in muscle, BAT or other white fat depots such as visceral or epididymal. During long-term experiments, BAT *FNDC5* mRNA expression levels increased proportionally to the percentage of fat mass. In WAT after three months with CR, *FNDC5* mRNA expression levels increased in epididymal and visceral adipose tissue but diminished with CR and HFD in subcutaneous fat, demonstrating differences in *FNDC5* expression depending on the adipose tissue depot. We suggest that the increase observed in epididymal and visceral WAT *FNDC5* expression, or at least partially, during CR occurs in an attempt to compensate for the decrease that occurs in the mass of those deposits, which were more affected by CR than the subcutaneous WAT^51^. This hypothesis is reinforced by the increase in *FNDC5* mRNA expression levels in all WAT depots in diabetic rats, concomitant with a significant decrease in their fat mass, which even disappears (data not shown). We suggest that circulating irisin levels are a reflection of the combination of irisin released from various fat depots and from muscle, and that when the amount of WAT decreases substantially, *FNDC5* levels in these depots increase to attempt to compensate for the tissue loss, which may explain some of the observed discrepancies between insulin levels and *FNDC5*/irisin, such as metformin treatment or long-term caloric restriction.

Hypothalamic *FNDC5* expression did not change with any of the tested diets suggesting that its regulation does not depend on alimentation/nutrition. In this work, we observed that an acute leptin treatment increased hypothalamic *FNDC5* transcript levels and diminished their levels in gastrocnemius, BAT and visceral adipose tissue correlating nicely with the observed decline in circulating irisin levels. However, these differences disappeared in rats that had fasted for 48 h suggesting that peripheral leptin regulates hypothalamic and peripheral *FNDC5* gene expression in a nutritional-dependent fashion. In a previous work, Rodriguez *et al*. observed that leptin administration was associated with increased gastrocnemius *FNDC5* transcript levels, a slight increase in circulating irisin in mice and downregulated *FNDC5* expression in murine differentiated subcutaneous adipocytes^52^. Additional studies are required to identify the source of the discrepancies between different experiments, including the experimental design and animal model.

Numerous studies have reported a positive association of muscle FNDC5/irisin with insulin resistance, speculating on the negative desensitizing effects of irisin on insulin action^16^,^34^,^53^. However, the results of our study also support a relationship between insulin production and the levels of circulating irisin. Acute treatments with exogenous irisin enhanced circulating insulin levels displaying a relationship between irisin and insulin production. Moreover, situations with low levels of insulin including fasting and CR and treatments with leptin, alloxan, metformin or insulin (which decreases endogenous insulin production) are associated with reduced *FNDC5* gene expression levels in muscle but also reduced circulating irisin levels in most cases. These results are consistent with a recent study demonstrating that serum irisin concentrations were lower in type 2 diabetes *mellitus* patients and increased after continuous subcutaneous insulin^54^.

Two recent works in mouse showed that metformin promotes *FNDC5* gene expression and irisin release from skeletal muscle^55^,^56^. The differences with respect to this work could be due to the species, mice *vs.* rats and/or also to the effects of the metformin in the weight and body composition. In our case, the treatment with metformin resulted in a sharp decrease in both body weight and fat mass gain, but not in the mentioned works where no variations were observed on body weight of wild-type mice treated with metformin. Supporting this theory Li *et al*. found that after 6 months of metformin treatment, there was a significant decrease in % fat mass, body weight and circulating irisin in polycystic ovary syndrome women^57^. However, to clarify this point further studies are needed, where the body weight gain do not change between controls and rats treated with metformin.

Doubts have been raised about circulating irisin, and there are limitations of the irisin assay^22^,^58^. We cannot rule out that the ELISA assays used in this study detected false signals from cross-reacting proteins. However, Jedrychowski *et al*. using tandem mass spectrometry have unequivocally shown that human irisin exists, circulates, and is regulated by exercise^23^. Moreover, they used immunoblotting of irisin plasma samples with a polyclonal antibody from the same commercial source as that used in the ELISA assays in this work and detected a band at ≈12 kDa, the predicted size of the irisin polypeptide^23^, suggesting that this antibody works efficiently. A strong point in our work is that several of the experiments, fasting and leptin and alloxan treatments are repeated and the results are replicable for *FNDC5* gene expression and serum irisin levels with rare exceptions in alloxan experiments that can be explained by the variable duration between the two treatments of alloxan.

In conclusion, *FNDC5* mRNA was expressed in high levels in muscles, brain and in the reproductive neuroendocrine axis. We demonstrated for the first time that there are differences in *FNDC5* expression depending on the WAT depot and when the amount of one of these depots decreases considerably, *FNDC5* mRNA expression increases, probably as a compensatory effect. Serum irisin levels diminish after a 48-h fast and with leptin, insulin and alloxan treatments, but no changes were observed during long-term experiments with different diets. We suggested that the combination of different depots of adipose tissue and skeletal muscle expression correlates with circulating irisin levels. On the other hand, hypothalamic *FNDC5* expression did not change for any of the tested diets suggesting that their regulation is not dependent on alimentation but was increased with leptin, insulin and metformin treatments. These results suggest that the regulation of central and peripheral *FNDC5*/irisin expression are different and have different functions. However, additional experiments are required to characterize such functions.

## Additional Information

**How to cite this article**: Varela-Rodríguez, B. M. *et al. FNDC5* expression and circulating irisin levels are modified by diet and hormonal conditions in hypothalamus, adipose tissue and muscle. *Sci. Rep.* **6**, 29898; doi: 10.1038/srep29898 (2016).

## Figures and Tables

**Table 1:** Effect of caloric restriction and high fat diet during 3 months on % fat mass, % lean mass, body weight gain, and plasma glucose, insulin, leptin and triglycerides levels.

	CR	ND	HFD
Fat mass (%)	2.1 ± 0.6 a	9.5 ± 0.5 b	18.8 ± 0.9 c
Lean mass (%)	80.3 ± 0.7 a	73.1 ± 0.7 b	66.2 ± 0.8 c
Body weight gain (g)	173.2 ± 4.8 a	306.8 ± 9.0 b	362.0 ± 7.3 c
Glucose (mg/dl)	72.0 ± 1.4 a	78.44 ± 1.0 b	139.3 ± 4.0 c
Insulin (ng/ml)	1.01 ± 0.1 a	3.2 ± 0.3 b	4.1 ± 0.3 c
Leptin (ng/ml)	3.3 ± 0.6 a	8.5 ± 1.0 b	15.7 ± 0.8 c
Tryglicerides (mg/dl)	64.1 ± 3.9 a	120.8 ± 6.2 b	148.1 ± 8.3 c

Values are expressed as mean ± SEM. Different letters indicate statistical differences (One-way ANOVA with *post hoc* Tukey test, a value of *P* < 0.05 was considered statistically significant). CR: caloric restriction (n = 10), ND: normal diet (n = 10) and HFD: high fat diet (n = 10). The animals were reared in these diets during three months after weaning.

**Table 2:** Effect of fasting during 48-h on body weight gain, plasma glucose, insulin and triglycerides levels and somatic indices.

	Body weight gain (g)	Glucose (mg/dl)	Insulin (ng/ml)	Triglycerides (mg/dl)	Somatic index. Liver (g/100 g BW)	Somatic index. Epididymal (g/100 g BW)	Somatic index. Visceral (g/100 g BW)
Fed	41.2 ± 2.5	120 ± 1.5	2.3 ± 0.3	162 ± 13	3.7 ± 0.1	0.96 ± 0.05	0.68 ± 0.04
Fast	11.6 ± 4.4*	91 ± 1.1*	0.44 ± 0.07*	68 ± 4.7*	2.6 ± 0.08*	0.88 ± 0.05	0.49 ± 0.05*

Values are expressed as mean ± SEM. *P < 0.05 *vs.* fed, t-test, n = 6. Somatic index was calculated as the ratio between tissues weight and body weight and was expressed as g/100 g body weight (BW).

**Table 3:** Effect of a 14-day IP saline and insulin and subcutaneous metformin treatment on body weight gain, and plasma glucose and triglycerides levels and somatic index.

	Body weight gain (g)	Glucose (mg/dl)	Triglycerides (mg/dl)	Somatic index. Liver (g/100 g BW)	Somatic index. Epididymal (g/100 g BW)	Somatic index. Visceral (g/100 g BW)
Control	50.0 ± 1.8 a	131.8 ± 4.7 a	116.6 ± 6.5 a	3.5 ± 0.1 a	1.57 ± 0.09 a	1.0 ± 0.06
Metformin	20.7 ± 7.4 b	91.0 ± 11.5 b	97.5 ± 10.5 a,b	3.3 ± 0.2 a,b	1.20 ± 0.03 b	0.9 ± 0.06
Insulin	41.4 ± 1.6 c	70.5 ± 10.5 b	69.76 ± 4.84 b	3.0 ± 0.1 b	1.63 ± 0.12 a	1.0 ± 0.04

Values are expressed as mean   SEM. Different letters indicate statistical differences by treatment (One-way ANOVA with *post hoc* Tukey test, a value of *P* < 0.05 was considered statistically significant). N = 6 (metformin treatment) or 8 (saline and insulin treatments). Somatic index was calculated as the ratio between tissues weight and body weight and was expressed as g/100 g body weight (BW).
